# Impact of a Mobile Telerehabilitation Solution on Metabolic Health Outcomes and Rehabilitation Adherence in Patients With Obesity: Randomized Controlled Trial

**DOI:** 10.2196/28242

**Published:** 2021-12-06

**Authors:** François Bughin, Gaspard Bui, Bronia Ayoub, Leo Blervaque, Didier Saey, Antoine Avignon, Jean Frédéric Brun, Nicolas Molinari, Pascal Pomies, Jacques Mercier, Fares Gouzi, Maurice Hayot

**Affiliations:** 1 PhyMedExp, Centre Hospitalier Universitaire Montpellier University of Montpellier Institut national de la santé et de la recherche médicale, Centre national de la recherche scientifique Montpellier France; 2 Centre de Recherche Institut Universitaire de Cardiologie et de Pneumologie de Québec Université Laval Québec, QC Canada; 3 Institut Desbrest de Santé Publique University of Montpellier Institut national de la santé et de la recherche médicale Montpellier France; 4 Endocrinologie-Diabétologie-Nutrition Centre Hospitalier Universitaire Montpellier University of Montpellier Montpellier France; 5 Institut Montpelliérain Alexander Grothendieck University of Montpellier Centre national de la recherche scientifique Montpellier France; 6 Department of Medical Statistics and Epidemiology Centre Hospitalier Universitaire Montpellier University of Montpellier Montpellier France

**Keywords:** telerehabilitation, mHealth, rehabilitation, obesity, mobile phone

## Abstract

**Background:**

Obesity is a major public health issue. Combining exercise training, nutrition, and therapeutic education in metabolic rehabilitation (MR) is recommended for obesity management. However, evidence from randomized controlled studies is lacking. In addition, MR is associated with poor patient adherence. Mobile health devices improve access to MR components.

**Objective:**

The aim of this study is to compare the changes in body composition, anthropometric parameters, exercise capacity, and quality of life (QOL) within 12 weeks of patients in the telerehabilitation (TR) program to those of usual care patients with obesity.

**Methods:**

This was a parallel-design randomized controlled study. In total, 50 patients with obesity (BMI>30 kg/m²) were included in a TR group (TRG) or a usual care group (UCG) for 12 weeks. Patients underwent biometric impedance analyses, metabolic exercise tests, actimetry, and QOL and satisfaction questionnaires. The primary outcome was the change in fat mass at 12 weeks from baseline. Secondary outcomes were changes in body weight, metabolic parameters, exercise capacity, QOL, patients’ adhesion, and satisfaction.

**Results:**

A total of 49 patients completed the study. No significant group × time interaction was found for fat mass (TRG: mean 1.7 kg, SD 2.6 kg; UCG: mean 1.2 kg, SD 2.4 kg; *P*=.48). Compared with the UCG, TRG patients tended to significantly improve their waist to hip ratios (TRG: −0.01 kg, SD 0.04; UCG: +0.01 kg, SD 0.06; *P*=.07) and improved QOL physical impact (TRG: +21.8, SD 43.6; UCG: −1.2, SD 15.4; *P*=.005). Significant time effects were observed for body composition, 6-minute walk test distance, exercise metabolism, sedentary time, and QOL. Adherence (95%) and satisfaction in the TRG were good.

**Conclusions:**

In adults with obesity, the TR program was not superior to usual care for improving body composition. However, TR was able to deliver full multidisciplinary rehabilitation to patients with obesity and improve some health outcomes. Given the patients’ adherence and satisfaction, pragmatic programs should consider mobile health devices to improve access to MR. Further studies are warranted to further establish the benefits that TR has over usual care.

**Trial Registration:**

ClinicalTrials.gov NCT03396666; http://clinicaltrials.gov/ct2/show/NCT03396666

## Introduction

### Background

Obesity is a chronic disease defined by a BMI of >30 kg/m² in the context of increased fat mass (FM). It is currently a highly prevalent disorder and a major public health issue [1]. It is associated with increased morbidity and mortality [2], including metabolic comorbidities, disabilities, and impaired quality of life (QOL). The metabolic risk is worst in cases of FM increase and when abdominal visceral fat predominates [3]. Thus, waist circumference (WC)—a marker of intraabdominal fat [4]—and the waist to hip ratio (WHR) better predict metabolic (ie, insulin sensitivity and lipid profile) and cardiovascular complications than BMI [5].

Obesity results in an imbalance between energy intake and energy expenditure [6,7]. Creating a negative energy balance can induce or maintain weight loss in patients with obesity [8]. Thus, physical activity (PA) and nutrition interventions are the cornerstones of obesity treatment, improving weight, WC, FM, and health outcomes [9]. Aerobic training alone induces significant weight loss in individuals with obesity. Specifically, light to moderate intensity corresponding to the intensity of the maximum lipid oxidation (LIPOXmax) individually determined in patients [10] has demonstrated significant weight, WC and FM reduction [8], as well as benefits on body composition and biological parameters (cholesterol and blood glucose) [11]. However, the most efficient strategy in obesity combines exercise training with nutrition interventions and therapeutic education [12,13] in a multidimensional metabolic rehabilitation (MR) for at least 12 weeks [14,15]. Although scientific societies recommend MR for patients with obesity [16], the benefits of such interventions remain to be compared with usual care alone.

However, the delivery of MR in the clinical field is a complex issue, and population-based trials have shown poor patient adherence (large dropout rates [17] and poor attendance [18]). This large underutilization of MR [19] is also because of the financial cost of such programs [20]. In the field of pulmonary rehabilitation, a widely developed domain, such barriers limiting the access to and delivery of rehabilitation have been well-described [21]. Thus, trials testing the effects of MR versus usual care—even if positive—would have limited clinical relevance because it is poorly applicable in patients with obesity.

The barriers of access to MR can be waived by recent technological innovations in the field of mobile devices. Mobile health (mHealth) facilities (smartphone-based educational apps, web-based tools, SMS text messaging, PDA physiological status monitoring, and connected captors) improved the delivery of the components of rehabilitation when taken individually [22]. In patients with obesity, a 10-week web-based exercise program has shown a significant effect on patients’ FM [23]. In addition, mHealth nutrition management or therapeutic education had significant effects on body weight (BW) and BMI in obesity [24,25]. Thus, because mHealth facilities deliver full MR, a telerehabilitation (TR) program could be more efficient than usual care in patients with obesity. In addition, this pragmatic research approach based on affordable tools could provide evidence for real-world MR.

### Objectives

Therefore, we developed a mobile TR solution for patients with obesity and used it in a blended multidisciplinary MR combining exercise training at LIPOXmax intensity, nutritional intervention, and educational tools. The aim of this randomized controlled study is to compare the changes in body composition, anthropometric parameters, exercise capacity, and QOL within 12 weeks of the TR program versus usual care in patients with obesity. In addition, feasibility, patients’ adherence satisfaction, and effects of this TR were assessed in the TR group (TRG).

## Methods

### Study Population

Adults aged 25-65 years with a BMI of ≥30 kg/m² were eligible for participation. The main exclusion criteria were participants with a contraindication for exercise training (such as unstable cardiovascular disease or musculoskeletal problems).

### Study Design

This was a 12-week, prospective, parallel-group, randomized controlled trial. Individuals were recruited from consultations of the Physiology Department of the University Hospital of Montpellier (France) and from the general population with media advertisements. After a screening period of 12 months, interested patients were contacted by email or phone and were registered on the Aviitam health platform. They were scheduled for half-day baseline assessments. All participants provided written informed consent. The study was conducted in accordance with the CONSORT (Consolidated Standards of Reporting Trials) ethical guidelines and the CONSORT of Electronic and mHealth Applications and Online Telehealth checklist [26]. The study was approved by the ethics committee (CPP Nord-Ouest IV, France; ClinicalTrials.gov identifier: NCT03396666).

Patients were admitted to the Physiology Department of the University Hospital of Montpellier (France) between January 2018 and November 2018. Baseline assessments included physical examination, bioimpedance, blood test, effort calorimetry, 6-minute walk test (6MWT), and self-questionnaires. Once baseline assessments were completed, participants were randomized to either a 12-week TR program or usual care. The randomization sequence was computer-generated using random blocks in an order unknown to investigators. The list was established by a statistician and was only accessible by the personnel in charge of randomization. Although participants could not be blinded to their treatment, both programs were presented as active interventions.

All tests and evaluations of the study were performed at the same place for each group under the same conditions and with the same devices. All assessments made at baseline were realized at the end of follow-up by technicians blinded to group allocation.

### Intervention and Control Groups

Patients from both groups had a specialized medical consultation with cardiopulmonary exercise testing and prescription of an adapted PA program at an intensity that elicited maximal lipid oxidation (called LIPOXmax). The patients in the usual care group (UCG) were advised to carry out their sessions independently, focusing on endurance PA sessions such as brisk walking, cycling, or swimming. Moreover, these patients received a booklet with different exercises and tips on PA and nutrition management. All patients were registered on the Aviitam website before and during the trial. Aviitam is a highly secure health record that allows the centralization, protection, and sharing of medical data with doctors. No restrictive diet was prescribed in either group.

**Figure 1:**
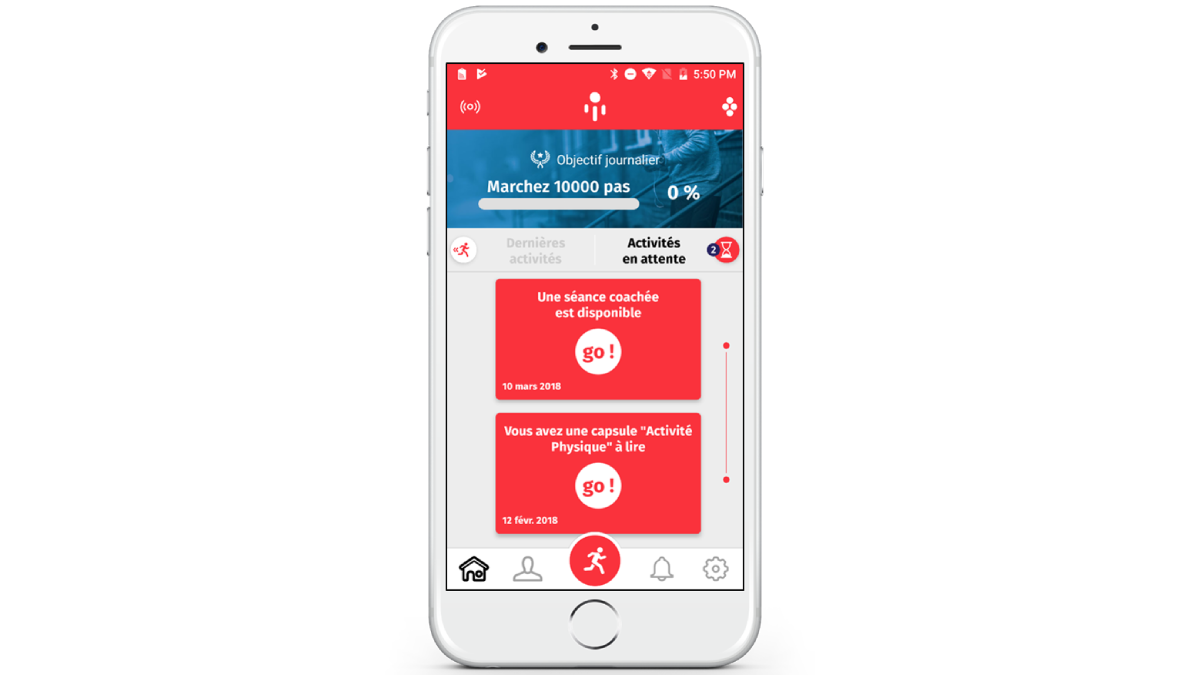
Telerehabilitation mobile app.

**Figure 2:**
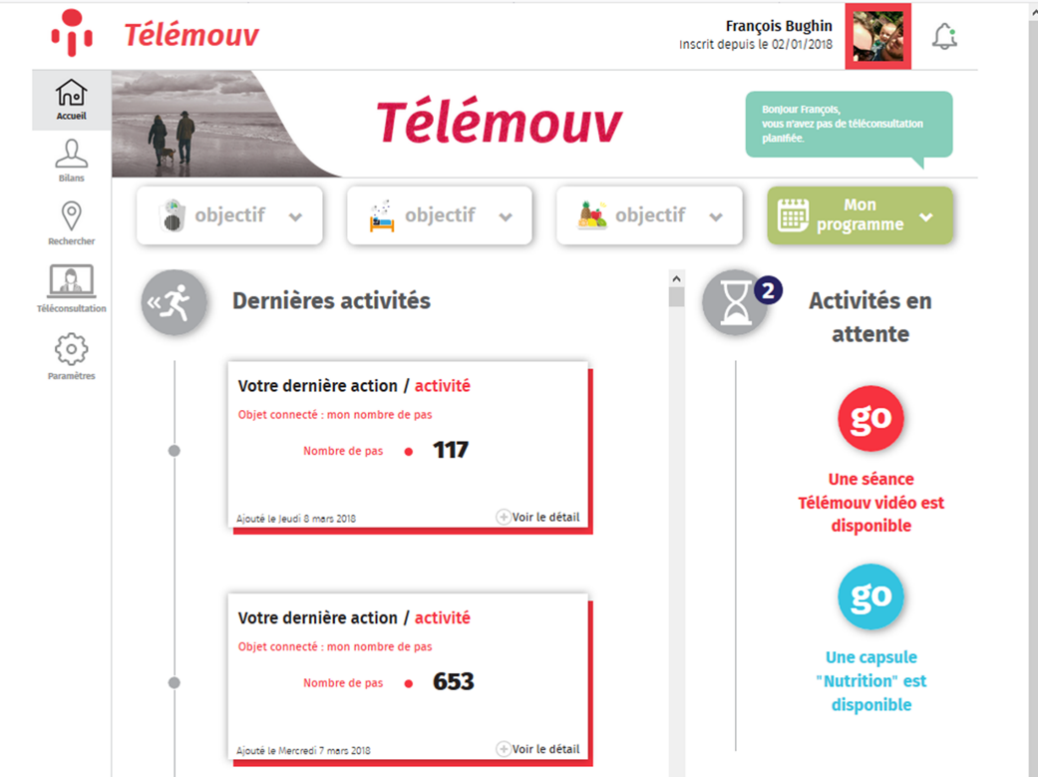
Telerehabilitation website.

The TR program is a multicomponent intervention available on smartphones (Figure 1) and the website (Figure 2). Patients received a package containing a smartphone (Archos with Android operating system) on which the TR Telemouv app was installed. A pedometer (Care Trackfit) and a heart rate monitor (Polar H7) were connected to the smartphone via Bluetooth. The patients received secure access codes for the app and website. They were trained in the use of the program and connected objects by a technician and received an instruction booklet to guide their first steps in the TR program. Telemouv solution contains 3 components: PA, nutritional, and educational programs. After 1 week, a PA teacher went to the patients’ homes to install a connected bicycle ergometer (Care Fitness) and performed the first supervised session. The exercise training program included endurance sessions, muscle reinforcement, and posture and balance exercises. Regarding endurance exercises, patients were advised to increase the volume and intensity of the sessions to reach the weekly goal of 150 minutes, with a minimum of 3 sessions per week, which could combine sessions on the connected cycloergometer and walking sessions. Wearing a connected heart rate monitor was recommended during endurance sessions to reach the target heart rate (corresponding to LIPOXmax). For muscle strengthening, balance, and posture exercises, patients had access to video sessions and were sent to the mobile solution throughout the program. Moreover, patients could track their daily step counts to reach their individualized goals. Nutrition management tools included hunger and satiety questionnaires and a 24-hour food intake questionnaire. Moreover, patients received daily educational content about illness, nutrition, and the benefits of PA. Patients with TRG had 2 teleconsultations at 1 and 2 months. In addition, doctors also had access to a secure website with access to patient data from the TRG.

### Outcomes

The primary outcome was the modification of the amount of FM, expressed in kilograms between baseline and 12-week follow-up in the TRG versus those in the UCG.

The main secondary outcomes were changes from baseline in body composition indexes (fat-free mass [FFM], muscular mass, and mass muscular index), anthropometric parameters (weight, BMI, WC, and WHR), and metabolism during exercise (maximal fat oxidation [MFO] and power at LIPOXmax and at crossover). Other outcomes were changes from baseline in PA and sedentary levels, exercise capacity, biological parameters, and self-assessment QOL questionnaires. The usability of the solution and satisfaction with the TR program were also assessed.

### Measures

Body composition; weight; height; and waist, hip, and neck circumferences were measured after 12 hours of fasting.

#### Bioelectrical Impedance Analyses

Participants’ body composition was assessed by bioimpedance analysis with a 6 terminal impedance plethysmograph (Biacorpus RX4000 software, BodyComp 8.4). This device measures the total resistance of the body to an alternating electric current of 50 kHz [27,28]. Body FM and FFM were calculated for each segment of the body according to the manufacturer’s database-derived disclosed equations and total water with published equations using the classical cylindrical model and Hanai mixture theory [29]. FM, FFM, and muscular mass were expressed in kilograms and as a percentage of total body mass. Muscle mass index was calculated as muscular mass/height² and expressed in kg/m².

#### Anthropometric Parameters

Height was measured to the nearest 0.5 cm using a standardized height gauge. BMI was calculated as weight (kg)/height² (m). Neck, chest, waist, and hip circumference measurements were obtained using standardized procedures. WHR was then calculated.

#### Metabolic Exercise Test

The participants performed an exercise test on an electromagnetically braked cycle ergometer (Ergoline Bosch 500, Ergoline) connected to a breath-by-breath device (COSMED Quark cardiopulmonary exercise testing, COSMED) for gas exchange measurements. The theoretical maximal aerobic power (W_max_ th) was calculated for all patients using Wasserman equations [30]. After a fasting period of 12 hours, participants underwent a standardized submaximal exercise test [31] consisting of five 6-minute submaximal steady-state workloads (set at 30%, 40%, 50%, and 60% of W_max_ th), with a calculation of carbohydrate and lipid oxidation rates from gas exchange measurements at steady state at the 5th to 6th minute of every step according to the nonprotein respiratory quotient technique [32]. Fat oxidation rates were calculated using the following equation:

Fat (mg/min) = −1.7012 × VCO_2_ + 1.6946 × VO_2_ (gas volume expressed in mL/min)

where, VCO_2_ is carbon dioxide output and VO_2_ is oxygen uptake.

After smoothing the curves, we calculated 2 parameters representative of the balance between fat and carbohydrate oxidation: the crossover point, which is the point at which carbohydrate becomes the predominant fuel representing more than 70% of the total energy [33] and the LIPOXmax point, where lipid oxidation reaches a maximum. The MFO rate is defined as the highest observed use of fat as an energy source during oxidative metabolism and is expressed in mg/min.

#### 6MWT Overview

The 6MWT was performed at the hospital on a plane surface in a 30-m–long covered corridor marked every 2 minutes. The tests were conducted according to the recommendations of the American Thoracic Society [34]. Heart rate and oxyhemoglobin saturation were recorded every minute, and dyspnea scores were measured on a Borg scale at the end of the test. The total distance was then recorded.

#### Questionnaires

QOL was assessed with a questionnaire for the general population (36-item short form survey, SF-36) and one specific to the population of patients with obesity (echelle qualité de vie, obésité et diététique [EQVOD]). The PA level was assessed using the Voorrips questionnaire (modified Baecke questionnaire). The questionnaire scored the past year’s household activities, sports activities, and other physically active leisure time activities and gave an overall PA score. The participants were asked to describe the type of activity, hours per week spent on it, and the period of the year in which the activity was normally performed. All activities were classified according to posture and movement. This questionnaire provides a reliable and valid method for classifying the activity level of older participants as high, medium, or low. With this method, normal participants with scores <9.4 are classified as having low PA.

The SF-36 is a generic self-reported measure of health-related QOL comprising 36 questions across 8 domains (physical functioning, role-physical, bodily pain, general health, vitality, social functioning, role-emotional, and mental health). Answers to each question are rated on a Likert-type scale and summed to produce a raw score that is transformed to a scale of 0 to 100, with higher scores indicating a better QOL [35]. SF-36 subscales were computed to generate 2 summary measures: the physical component summary and the mental component summary. EQVOD is a French, validated scale specific to obesity, derived from the Impact of Weight on QOL questionnaire and its short version Impact of Weight on QOL-Lite [36]. The EQVOD questionnaire was adapted to the sociocultural factors of obesity and its dietetic treatment in France. It is easy to self-administer.

Usability was evaluated with the System Usability Scale (SUS) [37]. It is a 10-item questionnaire with 5 response options for the respondents. An SUS score of >68 would be considered above average, and anything <68 is below average.

#### Blood Test

A venous blood sample was obtained in the fasting state to measure the lipid proﬁle, plasma glucose, insulinemia, and C-reactive protein.

#### Actigraphy

Participants wore a GT3X accelerometer (ActiGraph) on the nondominant wrist, programmed to collect data from the vertical axis in 15-second epochs and initialized using a normal filter (AGNorm). Accelerometers were worn for 7 days during all waking hours and removed for sleeping and during water-based activities. The minimum wearing criteria was ≥4 days, with ≥8 hours of wearing time each day [38]. In addition to the daily steps, daily sedentary time in minutes and daily time spent in moderate to very vigorous activity were extracted from actimetry according to the manufacturer’s specifications using the Freedson cutoff and the software (Actilife) provided by the company.

### Power Calculations and Statistical Analysis

The calculation of the number needed to treat is based on a hypothesis supported by the literature [8]. We expect a difference between the 2 arms of 1 kg of fat loss with a common SD of 1 kg. For an α threshold of .05 and a study power of 90%, the study included 22 patients in each group. Considering a possible 10% dropout rate, the study will need to include 25 patients by randomized arm to demonstrate an effect.

The baseline characteristics of the 2 groups were compared using the independent 1-tailed *t* test or Mann-Whitney U test according to the data distribution. The intra- and intergroup kinetics of changes for the variables under intervention were analyzed with linear mixed effects models including a time effect, a group effect, and the interaction between these effects as a fixed factor and a subject effect as a random factor, using the nmle package in R. In case of significant interaction effect, false discovery rate–adjusted post hoc tests were performed. Linear mixed effects assumptions were tested before each test. Per-group analysis of the effect of the intervention was performed in the TRG group using paired *t* tests. The effect size was also calculated using Cohen *d*. Spearman rank order or Pearson correlations, depending on the data distribution, were used to determine associations between continuous variables. The data were analyzed using R software (R 4.0.3; R Foundation for Statistical Computing) and plotted using Prism Software. Statistical significance was set at *P*<.05.

## Results

### Patients

**Figure 3:**
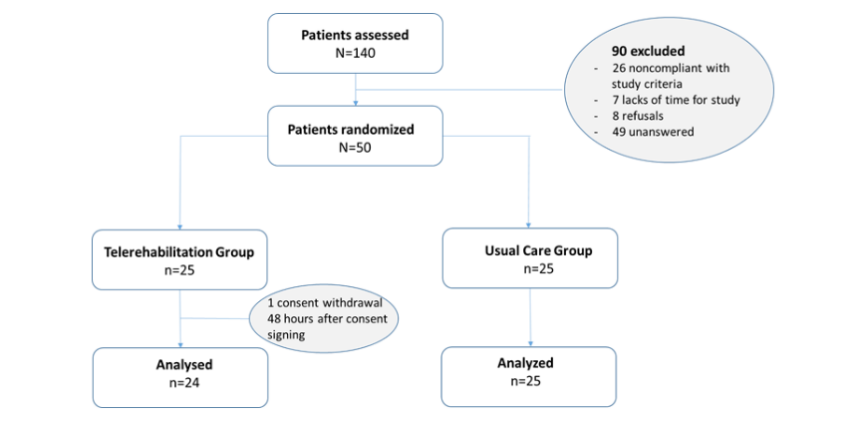
Study participant flowchart.

Of the 140 screened patients, 50 were included in the study and underwent randomization. A total of 49 patients completed the study (Figure 3). One patient in the TRG discontinued the trial before the endpoint without a postbaseline assessment. The baseline characteristics of both groups were not statistically different for any of the parameters assessed (Table 1). All severities were represented, as class I, II, and III obesity represented 44% (22/50), 36% (18/50), and 20% (10/50) of patients, respectively. A total of 90% (45/50) of patients had a low PA level, as defined by a Voorrips score of <9.4. None of the patients were currently medically treated for obesity. A total of 8 patients had diabetes, and 7 were treated for dyslipidemia.

**Table 1:** Characteristics of participants.

Characteristic	Intervention group (n=25)	Control group (n=25)	*P* value
Male, n (%)	11 (44)	12 (48)	.78
Age (years), mean (SD)	51.2 (10.8)	53.3 (11.3)	.47
Active smoker, n (%)	5 (20)	1 (4)	.23
Voorrips score, mean (SD)	5.7 (3.0)	6.4 (3.0)	.37
Total blood cholesterol (mmol/L), mean (SD)	5.2 (1.1)	5.1 (1.1)	.16
Fasting blood glucose (mmol/L), mean (SD)	7.3 (3.0)	5.9 (1.1)	.26
Fat mass (kg), mean (SD)	44.8 (10.6)	43.6 (18.1)	.51
Body weight (kg), mean (SD)	105.7 (18.1)	104.4 (14.9)	.78
BMI (kg/m²), mean (SD)	36.2 (4.2)	36.82 (5.0)	.74
Waist circumference (cm), mean (SD)	120.1 (11.5)	120.4 (14.1)	.21
Waist to hip ratio, mean (SD)	0.96 (0.07)	0.97 (0.10)	.05
6-minute walk test distance (m), mean (SD)	511 (69.6)	514.8 (69.9)	.77
LIPOXmax^a^ (W), mean (SD)	34.9 (11.6)	36.04 (10.3)	.87

^a^LIPOXmax: maximum lipid oxidation.

### Effects of TR on Primary and Secondary Outcomes

**Figure 4:**
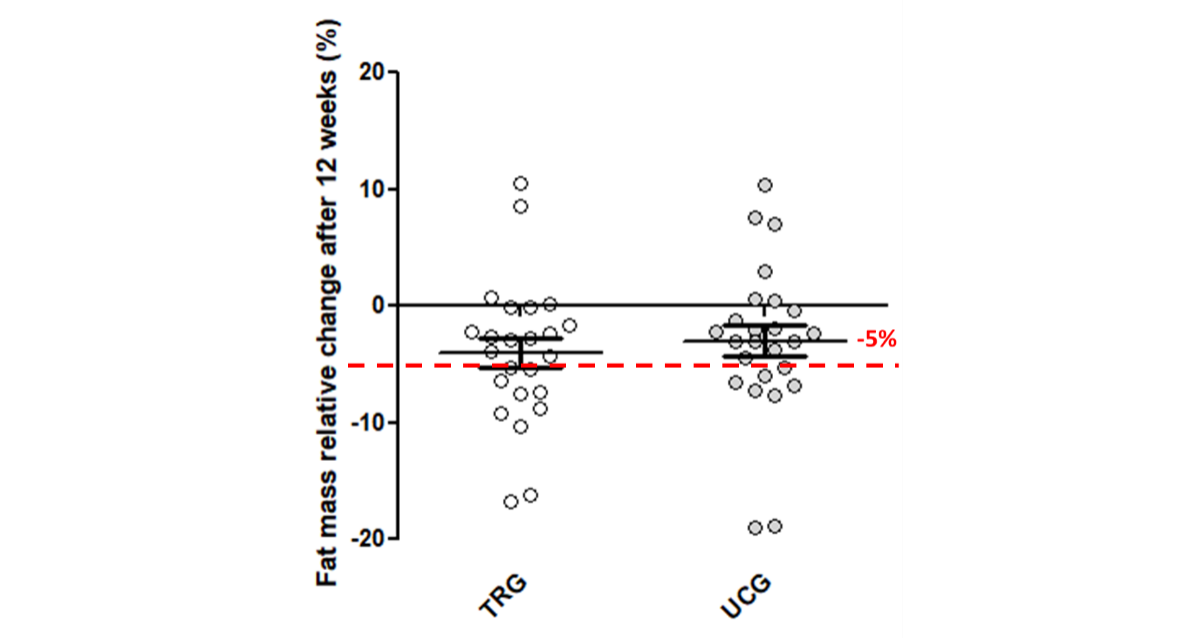
Relative change in fat mass after 12 weeks. TRG: telerehabilitation group; UCG: usual care group.

**Figure 5:**
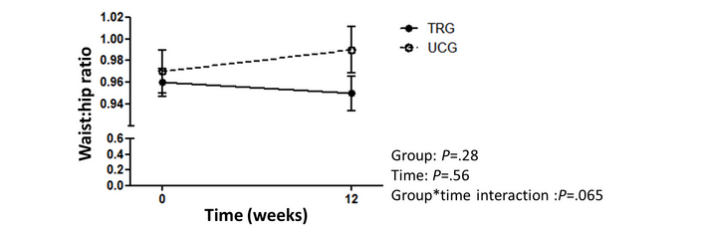
Waist to hip ratio change after 12 weeks (absolute). TRG: telerehabilitation group; UCG: usual care group.

**Figure 6:**
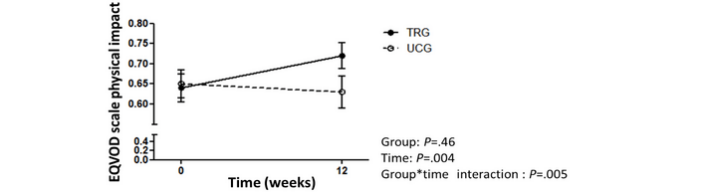
EQVOD scale scores for physical impact changes after 12 weeks (absolute). EQVOD: echelle qualité de vie, obésité et diététique; TRG: telerehabilitation group; UCG: usual care group.

No significant group or group × time interaction was found for the FM (Figure 2; Table 2). However, there was a significant time effect (*P*<.001), meaning that although not different between groups, an improvement in FM occurred in both study groups. An FM decrease was observed in the TRG (−1.7 kg, SD 2.6 kg; −4%, SD 6.2%) and in the in the UCG (−1.2 kg, SD 2.4 kg; −3%, SD 6.6%), with 48% (12/25) of patients improving the FM of >5% of initial values (Figure 4). Similarly, significant time effects, with no group × time interactions were observed for the FFM (%), muscle mass, 6MWT distance, crossover point, and power at the LIPOXmax and psychosocial component of the EQVOD (Table 2). Significant differences for a group × time interaction were found for the WHR (*P*=.07; Figure 5) and for the physical impact component of the EQVOD (*P*=.005; Figure 6), which was significantly increased in the TRG.

**Table 2:** Changes in primary and secondary outcomes between baseline and 12-week follow-up.

Outcome	Intervention group		Control group			*P* value (between group)		
	Baseline, mean (SD)	Follow-up, mean (SD)	Baseline, mean (SD)	Follow-up, mean (SD)	Group		Time	Interaction
Fat mass (kg)	44.80 (10.56)	43.18 (10.79)^a^	43.56 (12.19)	43.18 (13.18)^a^	.94		<.001	.48
Fat mass (%)	44.80 (10.56)	41.11 (7.50)^a^	41.88 (8.46)	41 (8.78)^a^	.86		<.001	.41
Fat-free mass (kg)	60.92 (13.35)	62.09 (14.08)	60.85 (11.75)	61.77 (12.03)	.95		.05	.45
Fat-free mass (%)	57.52 (7.17)	58.79 (7.52)	58.12 (8.46)	59 (8.78)	.84		<.001	.52
Muscle mass (kg)	26.99 (7.78)	28.23 (8.12)	27.33 (7.07)	27.98 (7.07)	.85		.02	.18
Muscle mass index (kg/m²)	9.10 (1.71)	9.49 (1.70)^a^	9.49 (1.83)	9.75 (1.95)	.39		.02	.26
Body weight (kg)	105.72 (18.06)	105.26 (19)	104.41 (14.86)	104.89 (16.69)	.89		.84	.41
BMI (kg/m²)	36.22 (4.15)	36.02 (4.40)	36.82 (5.00)	36.98 (5.72)	.54		.82	.41
Waist to hip ratio	0.96 (0.07)	0.95 (0.08)	0.97 (0.10)	0.99 (0.11)	.28		.56	.07
6-minute walk test distance (m)	511(70)	526 (71)	515 (70)	526 (67)	.90		.03	.75
Crossover point	63.96 (20.69)	72.96 (22.60)	66.48 (23.97)	71.16 (21.73)	.91		.002	.33
Power at crossover (W)	44.00 (17.16)	49.21 (16.52)	46.48 (14.21)	50.88 (18.10)	.67		<.001	.64
Power at LIPOXmax^b^ (W)	34.92 (11.59)	36.88 (11.51)	36.04 (10.33)	38.12 (13.31)	.75		.02	.90
MFO^c^ (mg/min)	270.5 (95.3)	298.4 (81.5)	301.5 (109.1)	303.9 (102.0)	.46		.14	.20
MFO (mg/min/kg FFM^d^)	10.61 (4.08)	11.21 (3.11)	11.23 (3.64)	11.20 (3.45)	.70		.52	.47
SF-36^e^ mental component	44.44 (12.43)	48.29 (10.06)	43.74 (11.97)	43.71 (12.89)	.48		.92	.13
SF-36 physical component	47.92 (7.31)	45.90 (8.46)	43.96 (9.23)	45.49 (10.18)	.26		.08	.34
EQVOD^f^ physical impact	64 (17)	72 (16)^a^	72 (16)	72 (16)	.46		.004	.005
EQVOD psychosocial	68 (19)	75 (21)	64 (21)	66 (22)	.18		.01	.17

^a^*P*<.05 between baseline and follow-up (within group).

^b^LIPOXmax: maximum lipid oxidation.

^c^MFO: maximal fat oxidation.

^d^FFM: fat-free mass.

^e^SF-36: 36-item short form survey.

^f^EQVOD: echelle qualité de vie, obésité et diététique.

### Effect and Feasibility of the TR in Users

TR patients who completed the 12-week intervention performed an average of 30.5 (SD 16.7) sessions of PA and connected 283.5 (SD 193.4) times to the app and the website throughout the study. The SUS score averaged 65.3 (SD 15.02). A score of >68, indicating good usability of the system, was found in 46% (11/24) of patients. Only 1 patient found the program with poor usability, with an SUS score of <39. In the TRG, per-group analyses showed an improvement in body composition, exercise metabolism, and health-related QOL after the intervention. The FM (−1.7 kg, SD 2.6 kg; *P*=.004), FFM (+1.2%, SD 1.9%; *P*=.005), muscle mass (+0.9 kg, SD 2.0 kg; *P*=.03), crossover point (+8.6, SD 14.0; *P*=.006), power at the crossover point and LIPOXmax (+5.8 W, SD 9.4 W, *P*=.006 and +2.48, SD 5.2 W, *P*=.03, respectively), SF-36 mental component (+3.53, SD 7.19; *P*=.04), and EQVOD physical and psychosocial components (+9.4, SD 14.1, *P*=.005 and +6.4, SD 11.7, *P*=.02, respectively) showed statistically significant improvements. Effect sizes ranged from small to moderate according to Cohen *d* (Table 3).

**Table 3:** Changes in parameters in the telerehabilitation group (paired *t* test and Cohen *d*).

Parameter	T0 (n=25), mean (SD)	T12 (n=25), mean (SD)	Difference (n=25), mean (SD)	*P* value	Effect size (Cohen *d*)	Magnitude
Fat mass (kg)	44.80 (10.56)	43.17 (10.79)	−1.70 (2.60)	.004	−0.65	Small
Fat mass (%)	42.48 (7.17)	41.11 (7.50)	−1.31 (1.94)	.003	−0.67	Moderate
Fat-free mass (kg)	60.92 (13.35)	62.09 (14.08)	0.86 (2.42)	.10	0.35	Moderate
Fat-free mass (%)	57.52 (7.17)	58.79 (7.52)	1.21 (1.89)	.005	0.64	Moderate
Muscle mass (kg)	26.99 (7.78)	28.23 (8.12)	0.94 (1.97)	.03	0.48	Small
Muscle mass index (kg/m²)	9.10 (1.71)	9.49 (1.70)	0.30 (0.61)	.03	0.49	Small
Body weight (kg)	105.72 (18.06)	105.26 (19.00)	−0.85 (2.82)	.16	−0.30	Small
BMI (kg/m²)	36.22 (4.15)	36.02 (4.40)	−0.31 (1.02)	.15	−0.31	Small
Waist to hip ratio	0.96 (0.07)	0.95 (0.08)	−0.01 (0.04)	.25	−0.24	Small
6-minute walk test distance (m)	511.00 (69.58)	526.46 (70.79)	13.88 (36.44)	.08	0.38	Small
Crossover point	63.96 (20.69)	72.96 (22.60)	8.58 (14.02)	.006	0.61	Moderate
Power at crossover point (W)	44.00 (17.16)	49.21 (16.52)	5.79 (9.43)	.006	0.61	Moderate
Power at LIPOXmax^a^ (W)	34.92 (11.59)	36.88 (11.51)	2.38 (5.15)	.03	0.46	Small
Maximal fat oxidation (mg/min)	270.48 (95.25)	298.38 (81.45)	25.88 (73.12)	.10	0.35	Small
SF-36^b^ mental component	44.44 (12.43)	48.29 (10.06)	3.53 (7.19)	.04	0.49	Small
SF-36 physical component	47.92 (7.31)	45.90 (8.46)	−1.44 (6.76)	.36	−0.21	Small
EQVOD^c^ physical impact	63.70 (17.45)	72.25 (16.11)	9.44 (14.11)	.005	0.67	Moderate
EQVOD psychosocial	67.94 (19.32)	75.28 (21.30)	6.35 (11.66)	.02	0.54	Moderate

^a^LIPOXmax: maximum lipid oxidation.

^b^SF-36: 36-item short form survey.

^c^EQVOD: echelle qualité de vie, obésité et diététique.

### Baseline and Intervention-Induced Change Correlations in Parameters

At baseline, univariate correlations between parameters were found in all patients with obesity. FM was correlated with BMI (*r*=0.850; *P*<.001). FFM and muscle mass were correlated with the crossover point (*r*=0.509, *P*<.001 and *r*=0.507, *P*<.001, respectively), LIPOXmax (*r*=0.495, *P*<.001 and *r*=0.469, *P*<.001, respectively), and MFO (*r*=0.365, *P*=.009 and *r*=0.34, *P*=.02, respectively). The 6MWT distance was correlated with muscle mass (*r*=0.316; *P*=.03), LIPOXmax (*r*=0.275; *P*=.05), and the SF-36 physical impact (*r*=0.301; *P*=.05). EQVOD’s psychosocial score was correlated with FM (*r*=−0.338; *P*=.02) and WHR (*r*=0.281; *P*=.05). In addition, at the end of the 12-week trial, the change in BW was correlated with relative changes in FM (%) and FFM (%) in the TRG and UCG (TRG: *r*=0.598, *P*<.001 and *r*=0.670, *P*<.001, respectively; UCG: *r*=0.616, *P*=.01 and *r*=0.426, *P*=.04, respectively). In the whole population (N=49), BW change was inversely correlated with the 6MWT distance (*r*=−0.281; *P*=.05). In contrast, the number of training sessions and changes in WHR or FM (*P*=.56 and *P*=.26, respectively) and the number of connections and changes in WHR or FM (*P*=.86 and *P*=.69, respectively) were not significantly correlated.

## Discussion

### Principal Findings

This study was one of the first to propose the use of a mobile TR program using mHealth devices to deliver full MR in patients with obesity. Although our study did not show significant additional benefits versus usual care regarding the primary outcome (FM), there was a significant advantage regarding the domain of QOL and tendency for the WHR. In addition, per-group analysis indicated that the significant time effects on body composition, exercise capacity, PA behavior, and QOL were mainly because of the TRG. These effects occurred while the TR solution’s adherence and usability were good during the 12-week trial duration.

### Comparison of the Effects of TR Versus Usual Care in Patients With Obesity

Scientific societies have recommended that patients with obesity should benefit from a multidisciplinary program including exercise, diet, and cognitive behavioral therapy [16]. However, this recommendation was not based on randomized controlled trial evidence, except for one study [39]. In patients with obesity, rehabilitation improvements in BW, exercise capacity, and comorbidities have not been compared with those of a control group [40-42]. Thus, our randomized controlled trial fills a gap in scientific knowledge regarding obesity treatment. Although our study did not show significant improvement in FM—the primary outcome of the study—with TR versus usual care, numerous observations have to be underlined. Among the secondary outcomes, we observed a significant time × group effect for the physical impact domain of the EQVOD. This result is in line with the improvement of SF-36–assessed QOL previously reported in patients with obesity by MR [43], particularly after 12 weeks [44]. There was also a tendency for the WHR in favor of the TRG versus UCG (*P*=.07), which is consistent with previous reports of the effect on WC induced by multidisciplinary rehabilitation [45] or 12-week exercise training [46]. This effect of the TR would be clinically relevant, given the critical role of the visceral fat accumulation in the patients’ comorbidities [3,47]. As power calculation was performed on the basis of expected change in FM, further studies are required to confirm the effect of the TR on these secondary outcomes. Nonetheless, the mixed model showed significant time effects for most of the secondary outcomes (FM [kg, %], FFM [%], muscle mass [kg], muscle mass index, 6MWT distance [m], crossover point, intensity at the LIPOXmax, sedentary time [%], SF-36, and EQVOD), which requires per-group analyses to complete the interpretation.

### Effects in the UCG

Detailed, structured PA counseling in daily life was provided to the UCG patients in line with the guidelines for obesity [48]. The intensity of the endurance exercises was set at LIPOXmax, an individual intensity determined on the metabolic exercise test, but there was no supervision. Therefore, UCG patients did not benefit from the metabolic effects reported after 8-12 weeks of supervised training at LIPOXmax intensity [8,49]. Similarly, patients also benefited from nutrition counseling through the Aviitam platform registration, but the nutritional intervention was not supervised. Thus, the metabolic effects were logically not significant and close to those reported in previous UCGs in obesity [50]. Altogether, this means that although optimized standard care with specific assessments, prescription, and counseling was provided to the UCG patients, its short-term impact on the patients’ metabolism was limited.

### Effects in the TRG

In contrast, patients in the TRG showed significant improvements in body composition (FM), exercise capacity (6MWT distance), exercise metabolism (intensity at the crossover point and LIPOXmax), health-related QOL (SF-36 and EQVOD), biological parameters (total and low-density lipoprotein cholesterol), and sedentary time in each group analysis (Multimedia Appendices 1 and 2). All these parameters constitute classical outcomes that are improved by multidisciplinary MR. The results showed internal validity because physiological correlations were found between study parameters at baseline (FFM and exercise metabolism, 6 MWT distance and muscle mass, LIPOXmax, and SF-36), and after 12 weeks. The magnitude of the BW loss after 3 months in the TRG (−2.82%, SD 2.81%) appeared to be limited. However, this is in line with the 6% BW loss after 12 months of nonsurgical clinical obesity services [17,39]. In addition, clinical benefits have been reported in randomized controlled trials reporting weight loss of ≤3%, when mediated by physical exercise [51], particularly regarding body composition [52]. Training combined with diet induced a 5.1% reduction in FM [53], and the 4% (SD 6%) FM decrease in our TRG was consistent with previous studies. The effect size for FM loss was medium (Cohen *d*>0.50) and reached 5% or more in 48% (12/25) of patients in the TRG. These effects appear to be particularly relevant, as FM loss appears to be the best predictor of physical functioning improvement during weight loss in patients with obesity [13]. In addition, FM loss has been associated with the improvement of systemic inflammation and lipid profiles [47,54]. The decrease in total and low-density lipoprotein cholesterol (Multimedia Appendix 2) in the TRG supports the clinical relevance of FM loss in the TRG. The absolute and relative increase in intensity at the crossover point was +17% (SD 27%), less than previously reported [49], but Cohen *d* was 0.644, which indicates a medium improvement exercise metabolism in patients with obesity. In addition, given that the minimally clinically important difference has been estimated from 2 to 4 points of the SF-36 physical component summary or mental component summary [55,56], 25% to 35% and 5% to 10% of the patients with obesity in the TRG experienced clinically significant improvements in the physical and mental components of QOL, respectively. Altogether, results in patients with obesity of the TRG support mild to medium effects of multidisciplinary TR, with benefits being clinically in a significant, relevant proportion of patients.

### mHealth to Foster Adherence to MR in Patients With Obesity

TR has been studied in several conditions such as stroke [57], chronic obstructive pulmonary disease [58], cardiac diseases [59,60], diabetes [61], or neurodegenerative diseases [62]. To the best of our knowledge, this study is the first to test a TR solution in a population of patients with obesity. Current mHealth interventions for obesity have been limited to self-management, self-monitoring, PA, or nutrition or education alone [63]. A meta-analysis of mHealth in obesity has shown heterogeneous evidence of health outcomes [25]. However, establishing the impact of mHealth-based MR was not the aim of our study. In accordance with previous studies, our strategy was to develop an mHealth device to improve access to MR in patients with obesity. Attendance and dropout represent a critical issue in rehabilitation because previous studies have shown that the highest clinical benefits were seen in participants or patients with obesity with the highest attendance [64,65]. Accordingly, we found that the adherence of the 12-week program reached 95% in the TRG, with only 1 dropout. This is largely above the dropout rate reported in mHealth studies [66]. Dropout rates usually range from 43% to 62% over 6-24 months during multidisciplinary rehabilitation for patients with obesity [17]. This high attendance in the TRG was in line with the SUS score showing that the solution was acceptable to the patients. Thus, our study showed that our TR solution succeeded in overcoming some of the barriers to face-to-face rehabilitation and appeared to be a relevant tool to deliver MR in patients with obesity.

### Study Limitations

One limitation of the study was the lack of sufficient objective monitoring of the intervention in the TRG. Patients experienced difficulties, mainly secondary to connectivity defects with Bluetooth, to use heart rate monitors, pedometers, and cycle ergometers. Therefore, too few data were collected to monitor the intervention correctly. However, our study was a pragmatic trial, and all analyses were intention-to-treat analyses, which means that the impact of these missing data on the results was limited. Nonetheless, monitoring the intervention would have allowed for a better understanding of the effects of the TRG in patients with obesity, particularly in terms of the patients’ phenotype in response to the intervention. In addition, the information technology firm that codeveloped the TR solution with our team did not have the opportunity to implement push notifications and provide pertinent feedback to patients. Finally, it is probable that patients need a longer and more stimulant intervention. Therefore, long-term intervention and the addition of human support, for example via videoconferencing, could address these limitations and improve the outcomes of TR programs. Altogether, the development of a TR solution remains an issue that must be addressed from the global perspective of our mHealth project.

### Conclusions

In adults with obesity, our TR program was able to deliver full MR but did not demonstrate superiority to the usual care on body composition. Over a period of 12 weeks, it induced effects on most rehabilitation outcomes in patients with obesity (body composition, total cholesterol, and lipid oxidation during exercise). These effects were not significantly superior to those induced in our UCG. However, the excellent patient adherence to the TR constitutes an answer to the challenge of patient adherence to face-to-face rehabilitation programs. Pragmatic MR programs should consider mHealth devices to deliver interventions. In parallel to the continuous development of technological solutions, large-scale and long-term studies are needed to translate these technological promises into fully efficient interventions in the clinical field.

## Appendix

Multimedia Appendix 1 Changes in activity level between baseline and 12-week follow-up in per-protocol population.

Multimedia Appendix 2 Changes in biological parameters between baseline and 12-week follow-up in per-protocol population.

Multimedia Appendix 3 CONSORT-eHEALTH checklist (V 1.6.1).

## Abbreviations

6MWT: 6-minute walk test
BW: body weight
CONSORT: Consolidated Standards of Reporting Trials
EQVOD: echelle qualité de vie, obésité et diététique
FFM: fat-free mass
FM: fat mass
LIPOXmax: maximum lipid oxidation
MFO: maximal fat oxidation
mHealth: mobile health
MR: metabolic rehabilitation
PA: physical activity
QOL: quality of life
SUS: System Usability Scale
TR: telerehabilitation
TRG: telerehabilitation group
UCG: usual care group
WC: waist circumference
WHR: waist to hip ratio
