# Supplementary figures and images for: Impact of a Mobile Telerehabilitation Solution on Metabolic Health Outcomes and Rehabilitation Adherence in Patients With Obesity: Randomized Controlled Trial
Source: JMIR Mhealth Uhealth. 2021 Dec 6;9(12):e28242. doi: 10.2196/28242 (PMC8691412; doi:10.2196/28242)

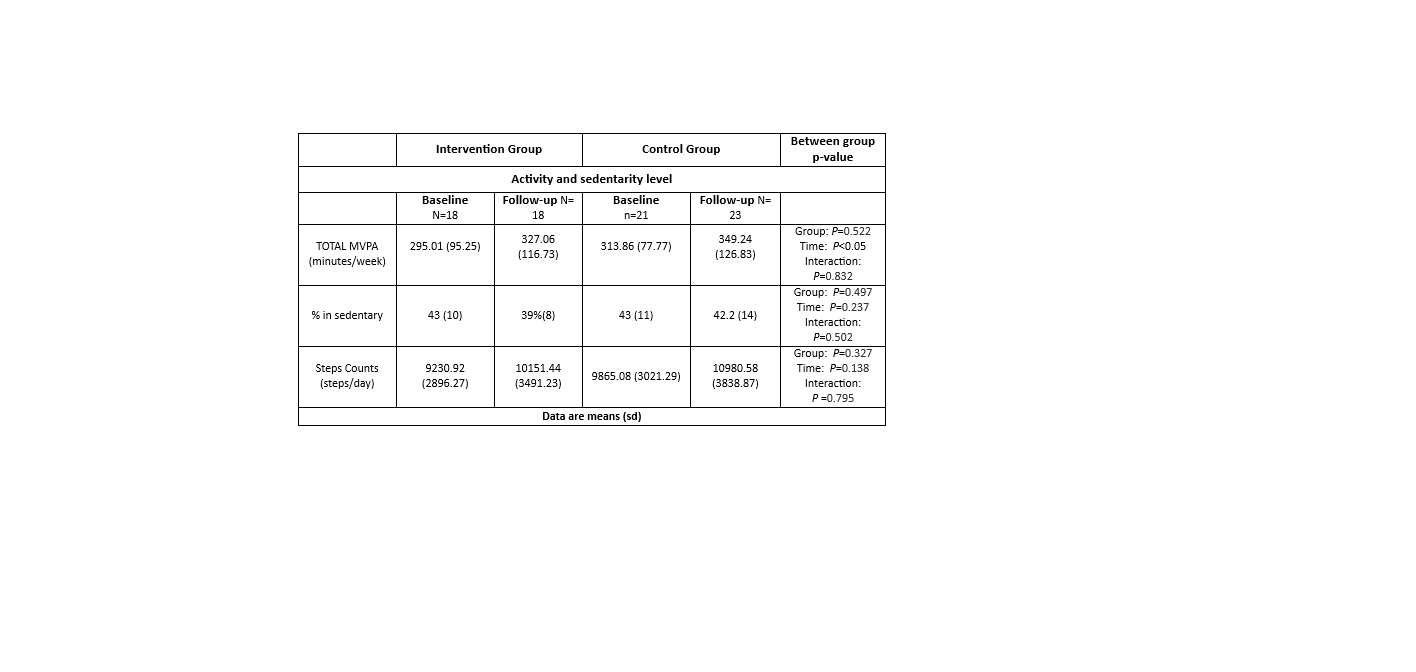

Supplement: Multimedia Appendix 1 [file mhealth_v9i12e28242_app1.png]

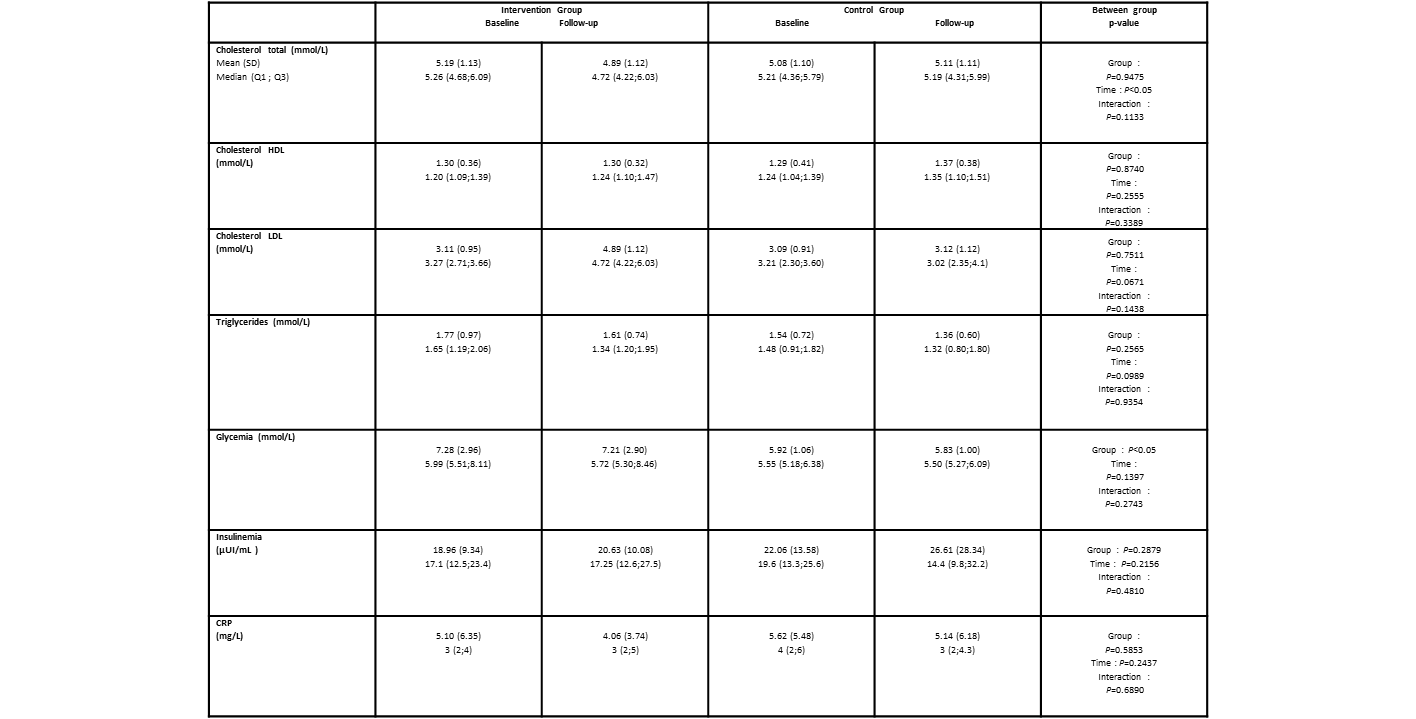

Supplement: Multimedia Appendix 2 [file mhealth_v9i12e28242_app2.png]
